# Prep1 (pKnox1) Regulates Mouse Embryonic HSC Cycling and Self-Renewal Affecting the Stat1-Sca1 IFN-Dependent Pathway

**DOI:** 10.1371/journal.pone.0107916

**Published:** 2014-09-18

**Authors:** Livia Modica, Giorgio Iotti, Annalisa D’Avola, Francesco Blasi

**Affiliations:** Istituto FIRC di Oncologia Molecolare (IFOM), Milan, Italy; Emory University, United States of America

## Abstract

A hypomorphic *Prep1* mutation results in embryonic lethality at late gestation with a pleiotropic embryonic phenotype that includes defects in all hematopoietic lineages. Reduced functionality of the hematopoietic stem cells (HSCs) compartment might be responsible for the hematopoietic phenotype observed at mid-gestation. In this paper we demonstrate that Prep1 regulates the number of HSCs in fetal livers (FLs), their clonogenic potential and their ability to *de novo* generate the hematopoietic system in ablated hosts. Furthermore, we show that Prep1 controls the self-renewal ability of the FL HSC compartment as demonstrated by serial transplantation experiments. The premature exhaustion of Prep1 mutant HSCs correlates with the reduced quiescent stem cell pool thus suggesting that Prep1 regulates the self-renewal ability by controlling the quiescence/proliferation balance. Finally, we show that in FL HSCs Prep1 absence induces the interferon signaling pathway leading to premature cycling and exhaustion of fetal HSCs.

## Introduction

Hematopoiesis is the production of blood cells in the embryo and throughout adult life. Hematopoietic stem cells (HSCs) produce and replace mature blood cells through self-renewal and differentiation. During embryonic development hematopoiesis occurs step-wise in different embryonic sites: in the yolk sac around E7, in the aorta-gonad mesonephros (AGM) at E10.5 [1] and from E11 in the fetal liver (FL). At mid-late gestation in FL, HSCs undergo a massive expansion generating the stem cell pool that will contribute to mature blood cells during entire life. Around birth HSCs move to the bone marrow (BM) where they reside mainly in a quiescent state during adult life [2]. Intrinsic factors, such as transcription factors and chromatin modifiers, and extrinsic microenvironmental factors encircling the HSCs modulate their activity during both embryonic and adult life [3]. The BM niche and the factors controlling adult HSCs have been extensively studied [4]–[9], but developmental mediators of HSCs biology remain largely unknown. Identifying the mechanisms regulating HSCs during development is crucial since often cells undergoing malignant transformation reacquire properties distinctive of stem cells during developmental stages [10]–[14]. Thus the molecular details of fetal HSCs may be critical for further elucidation of HSCs malignancy and possible new targets for cancer therapy. Finally, factors regulating expansion and proliferation of FL HSCs might help developing protocols for *ex vivo* expansion of HSCs for clinical applications.

Prep1 is a TALE family homeodomain transcription factor and plays an essential role in embryonic development [15]–[19]. *Prep1^−/−^* embryos die before gastrulation around E6.25 due to p53-dependent apoptosis of epiblast cells [19]. A hypomorphic *Prep1* (*Prep1^i^*) mutation that causes the expression of 3 to 10% of Prep1 protein induces later embryonic lethality around E17.5 [20]. However, one quarter of these hypomorphs escape embryonic death and are important for identifying adult biological functions of *Prep1*. Prep1 acts as a tumor suppressor controlling genome stability and is an essential regulator of hematopoietic differentiation [21]–[23]. Prep1 controls both myeloid and lymphoid differentiation as *Prep1^i/i^* FL cells generate less CFU-GEMM colonies and differentiate less B cells [24]. The thymus of adult *Prep1^i/i^* mice is underdeveloped and the T cell lineage is compromised [25]. All these features of *Prep1^i/i^* FL and BM can be reproduced by transplanting FL cells into ablated hosts. *Prep1^i/i^* FL cells compete inefficiently with wild-type (wt) cells in competitive repopulation assays suggesting a defect in the HSC compartment [24]. However, how Prep1 affects HSCs biology and which HSCs properties are regulated remain unanswered.

We now demonstrate that Prep1 regulates the number of HSCs in FLs as well as their functionality to *de novo* generate the hematopoietic system in ablated hosts. Prep1 controls self-renewal of the FL HSC compartment and governs the balance between quiescent and proliferating fetal HSCs. The absence of Prep1 derepresses the interferon (IFN) signaling pathway, Stat1 is activated and FL HSCs cycle more frequently. We also identify Prep1 target genes likely mediating these effects.

## Methods

### Mice

Prep1^i/i^ have been described [23], [26]. Congenic CD45.1 mice (B6.SJL-Ptprc Pepc/BoyCrl; Ly5.1) were purchased from Charles River. All animal experiments were performed in accordance with Institutional Animal Care and Use Committee of IFOM and approved by the Italian Ministry of Health (project #110/11). Animals were kept on a 12/12-h light/dark cycle (lights on at 07∶00 h) with free access to food and water. All animal handlings (transplantation, sacrifice etc.,) were in accordance with the guidelines established by EU (directive 2010/63/EU). Topical anesthesia (ELMA cream) applied to the site 30 minutes prior to intravenous injection of cells. Mice were sacrificed by carbon dioxide euthanasia.

### Cells

Cell suspensions were obtained from E14.5 FLs as already described [27]. BM cells were harvested by flushing femurs and tibias in PBS with 2% heat inactivated fetal bovine serum (staining buffer). Peripheral blood (PB) was obtained by tail bleeding. EML1 cells were the kind gift of Dr. Schickwann Tsai and were cultured as described [28]. pLKO.1 vectors encoding shRNA targeting murine Prep1 or nontargeting control were obtained from Sigma (St Louis, MO). Lentiviral infection was obtained by 2 cycles of spinoculation (1800 rpm, 45 minutes per cycle).

### Flow cytometry

Stainings of surface markers were performed in staining buffer with the following conjugated monoclonal antibodies purchased from BD (BD Pharmingen) or eBioscience: Gr1 (RB6-8C5), B220 (RA3-6B2), Ter119 (TER-119), CD3e (145-2C11), Mac1 (M1/70), Sca1 (D7 or E13–161.7), cKit (2B8), AA4.1 (AA4.1), CD41 (MWReg30), CD48 (HM48-I), CD150 (mShad150), Il7R (SB/199), CD45.2 (104), CD45.1 (A20). The lineage cocktail (Lin) was composed of Gr1, B220, CD3e and Ter119. Mac1 was included only in the Lin used for BM cells. Stainings of intracellular markers were performed as follows: after surface marker staining, cells were fixed and permeabilised by Cytofix/Cytoperm kit (BD) and stained for pSTAT1 (#9271, Cell signaling) or Ki67 (#51-36524, BD) for 45 min. Then, fluorochrome-conjugated secondary antibodies were added. For cell cycle staining Hoechst33342 (1 µg/mL, Sigma) was added to stain DNA. Apoptosis staining was performed using AnnxinV Binding Buffer (BD), FITC-AnnexinV (Sigma) according to the manufacture’s protocol. Stained cells were analyzed by FACSCalibur (BD Biosciences) or FACSCantoII (BD Biosciences). Cell sorting was performed by FACSAria (BD Biosciences). CellQuest, Diva and FlowJo (Tree Star) software were used for data acquisition and analyses.

### Long Term Culture-Initiating Cell (LTC-IC) assay

A feeder layer of freshly isolated wt BM cells was established plating 3×10^5^ cells/well (96 well plate). Cells were cultured in MyeloCult (Stem Cell Technologies) supplemented with 10^−6^ M Hydrocortisone (Sigma). Proliferation was blocked by X-Ray irradiation (15 gray) at 70–80% confluency. HSCs purified from FLs were plated on the established feeder layer as specified in the text. Cultures were kept for 5 weeks in MyeloCult and then transferred into 35 mm plates in methylcellulose (MethoCult, Stem Cell Technologies). Colonies were scored after 12 days.

### Transplantation assays

8 to 12 weeks old CD45.1 mice were lethally irradiated with a 6.5 gray dose of X-rays (experimentally determined as minimal lethal dose). Four hours after irradiation, unfractionated or purified FL cells (CD45.2) in competition with BM cells were injected into the tail vain of CD45.1 mice. The injected cell doses are indicated in the text for each transplantation. For secondary and tertiary transplantations, 2×10^6^ unfractionated BM cells were collected from recipients and injected into the tail vain of new hosts. Repopulation activity was evaluated by repopulating units (RU) as follows: RU = (%chimerism)×(n. competitors cells/10^5^)/(100–%chimerism) [29].

### Real-Time quantitative PCR

HSCs were sorted from Prep1^+/+^ and Prep1^i/i^ FLs and kept on ice. Cell lysis, RNA extraction, reverse transcription and pre-amplification were performed by Taqman kit #4387299 (Life Technologies, Carlsbad, CA) according to the manufacturer’s protocol. Taqman Assays-on-demand were purchased from Life Technologies.

Each PCR reaction was run in triplicate and GAPDH was used as housekeeping gene. Real-time PCRs were carried out on the ABI/Prism 7900 HT Sequence Detector System (Applied Biosystems, Foster City, CA).

### Statistical analysis

Values are expressed as mean and error bars represent SEM. The significance of differences was determined by two-tailed Student’s t test. Pvalue (p) ≤0.05 were considered significant (*p≤0.05; **p≤0.01; ***p≤0.001).

## Results

### Prep1 regulates the number of functional HSCs in E14.5 FLs

**Figure 1:**
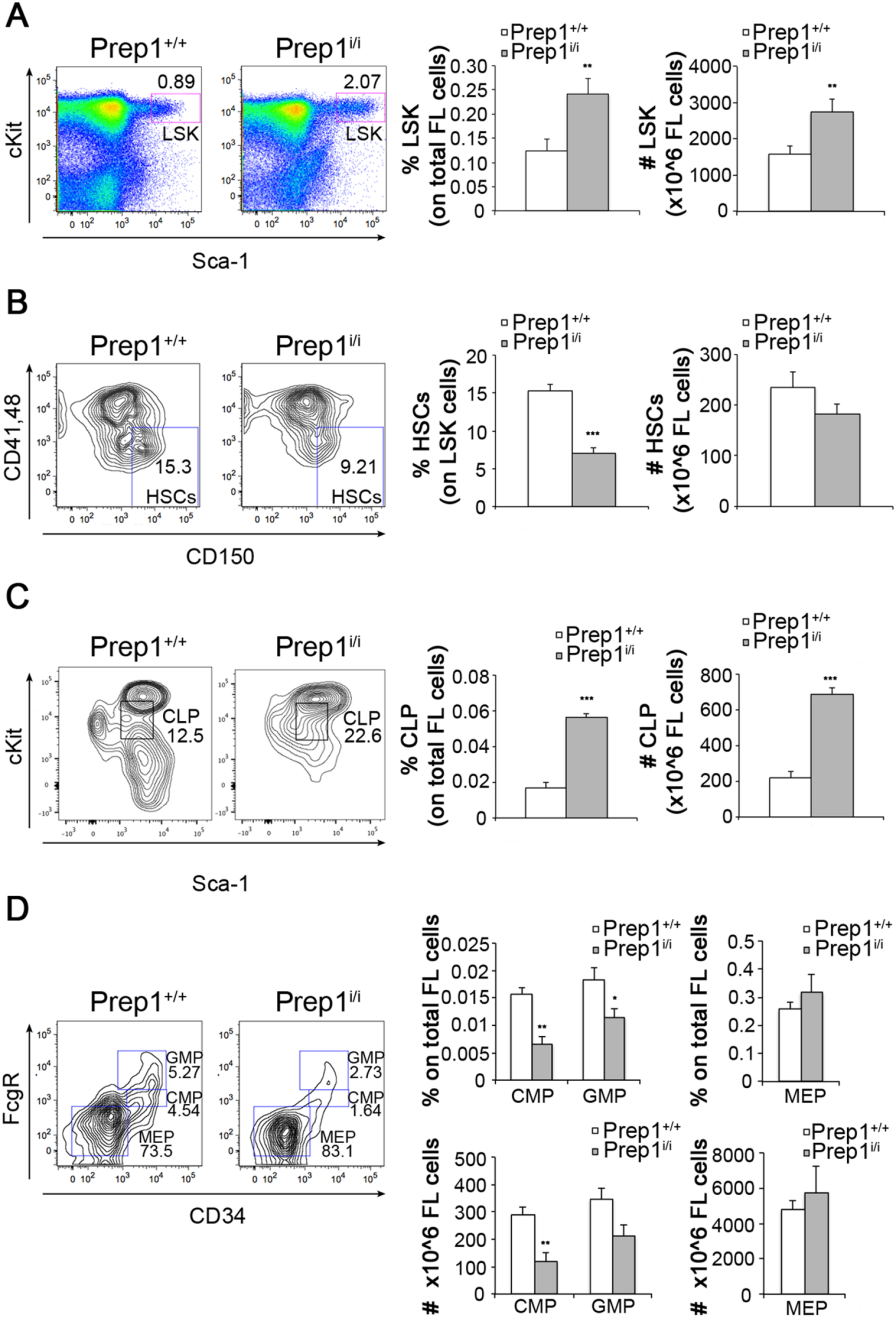
Prep1 affects stem and progenitors compartments in E14.5 FLs. (A–D) Representative FACS analyses of *Prep1^+/+^* and *Prep1^i/i^* FLs are shown on the left. Graphs describing percentage and absolute numbers are reported on the right. Numbers in the FACS plots indicate the percentage of cells in parental gates. (A) Lin^−^Sca1^+^cKit^+^ population is shown. FACS plots are referred to Lineage^−^ gate (n = 8 for each genotype; % L^−^S^+^K^+^ p = 0.01; # L^−^S^+^K^+^ p = 0.01). (B) HSC population is identified as L^−^S^+^K^+^CD150^+^ CD41^−^CD48^−^. FACS plots are referred to L^−^S^+^K^+^ gate (n = 8 for each genotype; % HSCs p = 0.0000056; # HSCs p = not significant). (C) CLPs are identified as Lin^−^Il7R^+^Sca1^int^ckit^int^. FACS plots are referred to Lin^−^Il7R^+^ gate. (n = 4 for each genotype; % CLPs p = 0.00014; # CLPs p = 0.00023). (D) FACS plots regarding myeloid progenitors refer to Lin^−^Sca1^−^ckit^hi^ gate. GMPs are FcγR^+^CD34^+^ (n = 4 for each genotype; % GMPs p = 0.05; # GMPs p = 0.07); CMPs are FcγR^int^CD34^+/l^°^w^ (n = 4 for each genotype; % CMPs p = 0.005; # CMPs p = 0.007); MEPs are FcγR^−^CD34^−^ (n = 4 for each genotype; % MEPs p = not significant; # MEPs p = not significant).

To investigate how Prep1 regulates the stem and progenitor compartments during development, we used E14.5 *Prep1^i/i^* FL cells. Initially we determined the immunophenotype of stem and progenitors populations in *Prep1^i/i^* FLs in comparison to wt controls. The Lin^−^Sca1^+^cKit^+^ (L^−^S^+^K^+^) population, mainly enriched in progenitors and to a lesser extent in stem cells, showed a 2-fold increase in Prep1 deficient embryos (Figure 1A). Further dissection of the L^−^S^+^K^+^ population with the CD150, CD48 and CD41 markers revealed a 50% reduction in the frequency of *Prep1^i/i^* L^−^S^+^K^+^ cells that express stem markers (CD150^+^CD48^−^CD41^−^) compared to the wt counterpart (Figure 1B). From now on and throughout the paper we will refer to the L^−^S^+^K^+^CD150^+^CD48^−^CD41^−^ cells as HSC as they represent an almost pure HSC population [30]. The absolute number of the *Prep1^i/i^* HSCs was similar to the wt because of the relative expansion of the L^−^S^+^K^+^ population (Figure 1B). Importantly Prep1 is expressed in the HSC compartment (see below).

We also analyzed lymphoid and myeloid committed progenitor populations. *Prep1^i/i^* common lymphoid progenitors (CLPs) showed a statistically significant 3.3 fold increase compared to wt controls (Figure 1C). Conversely, common myeloid progenitors (CMPs) and granulocyte/macrophage progenitors (GMPs) were reduced in frequency and absolute number when Prep1 was underexpressed (58% and 38% reduction, respectively) (Figure 1D). These data indicate a role for Prep1 in regulating the first steps of the hematopoietic differentiation during fetal development, from stem cells to lineage-primed progenitors, and that Prep1 exerts a peculiar differentiation stage-specific function.

**Figure 2:**
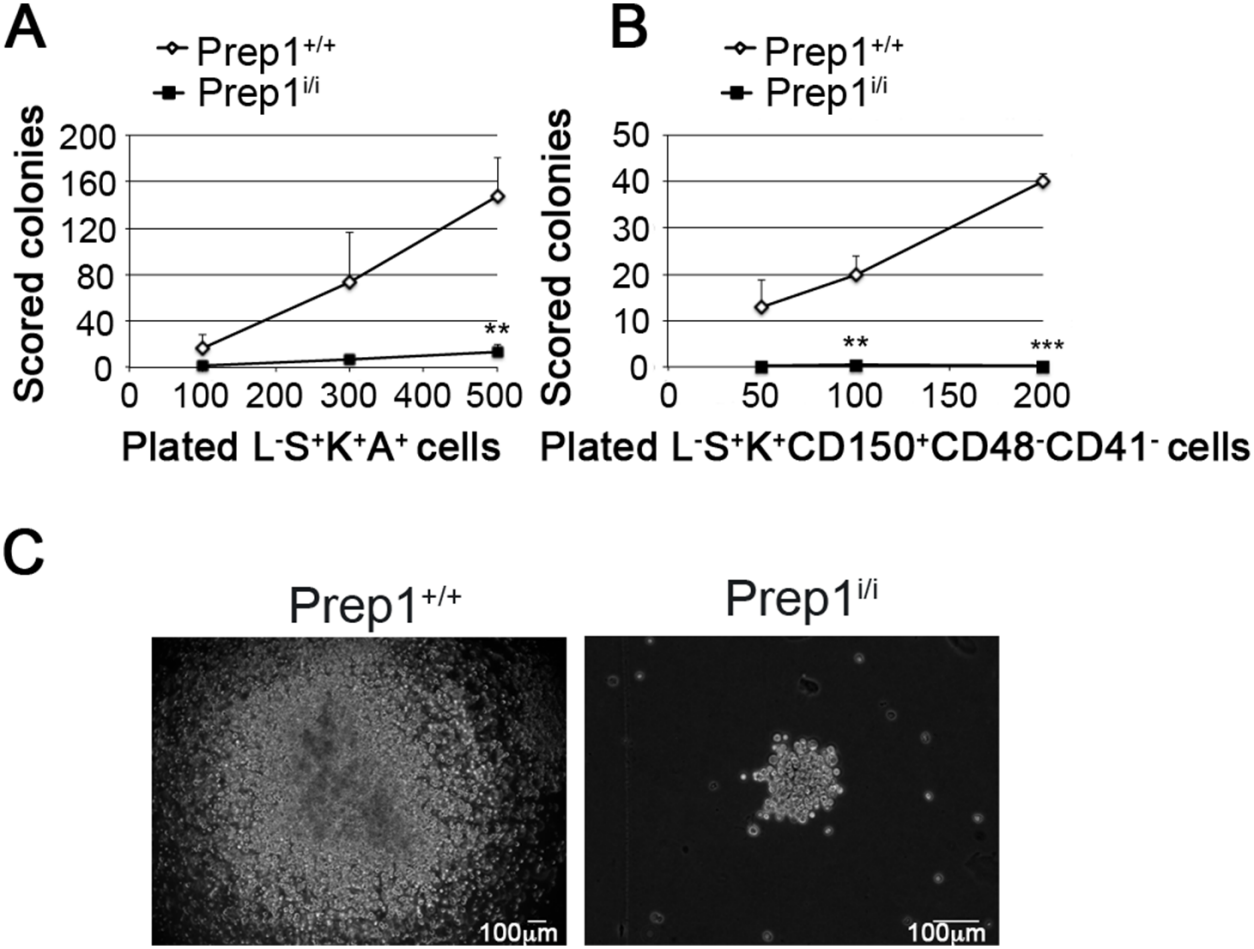
Impaired clonogenic activity of Prep1 deficient stem cell populations. (A) LTC-IC assay was performed plating 100, 300 or 500 L^−^S^+^K^+^A^+^ cells purified from *Prep1^+/+^* and *Prep1^i/i^* FLs. Mean value of scored colonies after 12 days in methylcellulose at each cell dose are shown (n = 9 at each cell dose for each genotype; 500 L^−^S^+^K^+^A^+^ p = 0.003, 300 and 100 L^−^S^+^K^+^A^+^ p = not significant). (B) LTC-IC was performed with 50, 100, 200 L^−^S^+^K^+^CD150^+^ CD48^−^CD41^−^ from both genotypes and colonies scored after 12 days in methylcellulose. (n = 4 at each cell dose for each genotype; 200 cells p = 0.001, 100 cells p = 0.01, 50 cells p = not significant). (C) Representative images of LTC-IC colonies obtained from *Prep1^+/+^* and *Prep1^i/i^* L^−^S^+^K^+^A^+^ cells (*Prep1^+/+^* colony: 4x original magnification; *Prep1^i/i^* colony: 10x original magnification; scale bars = 100 µm).

To functionally characterize the impact of Prep1 on HSCs we investigated their ability to form colonies *in vitro* in LTC-IC assay. We tested the HSC population (Fig. 1B) and the L^−^S^+^K^+^AA4.1^+^ cells (Figure S1). The latter is another cell population enriched for HSCs [31], [32]. Comparing the numbers of scored colonies at all plated cell doses, a strong decrease in the clonogenic potential of *Prep1^i/i^* stem/progenitor cells was observed (figures 2A and 2B). This agrees with our previous finding that myeloid, erythroid and immature CFU colonies were reduced in the absence of Prep1 [24]. Hypomorphic *Prep1^i/i^* generated smaller colonies in comparison to wt cells (Figure 2C). Thus, Prep1 acts on the functionality of stem/progenitor cells.

**Figure 3:**
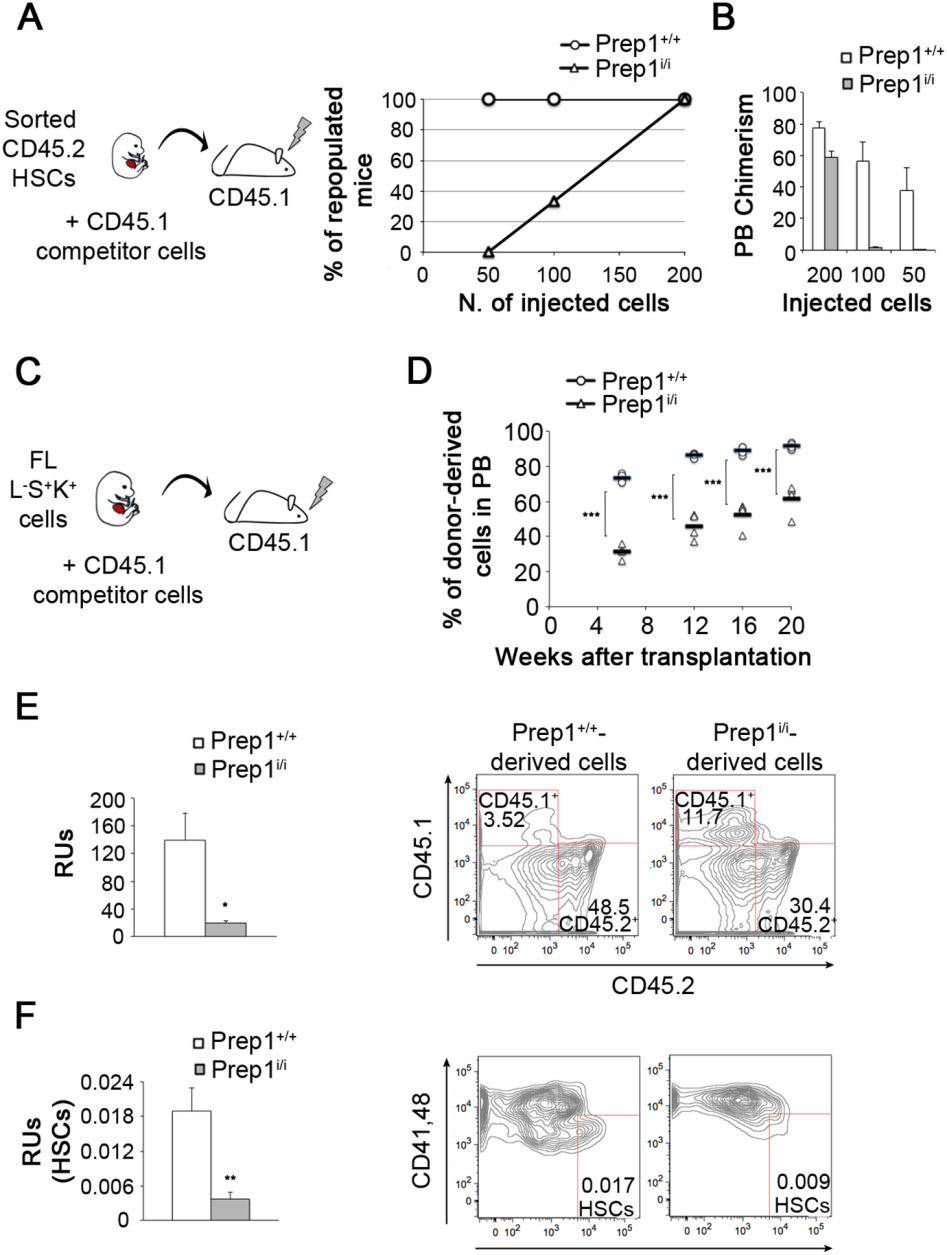
Prep1 controls the number and the functionality of HSCs in E14.5 FLs. (A) 50, 100 or 200 HSCs sorted from *Prep1^+/+^* and *Prep1^i/i^* FLs were transplanted into lethally irradiated mice in competition with 2×10^5^ CD45.1 BM cells. Mice showing more than 2% CD45.2^+^ cells in the PB were considered as positively repopulated. HSCs are identified as CD45.2^+^ L^−^S^+^K^+^CD150^+^CD48^−^CD41^−^cells. The graph represents the percentage of positively repopulated mice 16 weeks after transplantation (n = 4 for each genotype) (B) The graph indicates the mean chimerism shown by transplanted mice at each cell dosage in the PB 16 weeks after transplantation. (C) 2000 LSK cells purified form *Prep1^+/+^* or *Prep1^i/i^* FLs were transplanted in competition with 1×10^6^ BM cells into lethally irradiated CD45.1 recipients. (D) PB analyses to detect donor-derived (CD45.2^+^) cells performed at 7, 12,16 and 20 weeks after transplantation. In the graph, black bars represent the mean of CD45.2^+^ cells in the PB of *Prep1^+/+^* or *Prep1^i/i^* reconstituted mice (n = 4 for each genotype; p<0.001). (E–F) RUs (on the left) were calculated on FACS data (on the right) obtained from BMs of repopulated mice 20 weeks after transplantation. (E) Mean of RUs in Prep1^+/+^ or Prep1^i/i^-transplanted mice; p = 0.05. (F) Mean of HSCs RUs in *Prep1^+/+^* or *Prep1^i/i^*-transplanted mice; p = 0.01. HSCs are identified as CD45.2^+^ L^−^S^+^K^+^CD150^+^CD48^−^CD41^−^cells.

To investigate whether the reduced frequency and clonogenic potential of *Prep1^i/i^* HSCs depends on the lower number of HSCs, we analyzed the frequency of functional stem cells by an *in vivo* transplantation experiments with purified HSCs (Figure 3A). Wt HSCs repopulated all the recipients already at the lowest cell dosage (50 cells) whereas *Prep1^i/i^* HSCs fully repopulated recipients only at the highest dosages (Figure 3A; mice showing more than 2% donor-derived cells in the PB were considered as positively repopulated). The mean chimerism shown by transplanted mice at each cell dosage is shown in Figure 3B.

This set of experiments demonstrates that Prep1 controls the number of HSCs in FL and points to a role for this transcription factor in regulating also their functionality.

### Prep1 affects HSCs self-renewal by controlling their proliferation state

To analyze the role of Prep1 in regulating the functionality of fetal HSCs, we assessed the ability of Prep1 deficient cells to *de novo* generate the stem/progenitor compartments *in vivo*. We transplanted CD45.2 *Prep1^i/i^* or wt FL L^−^S^+^K^+^ cells in competition with wt CD45.1 BM cells (Figure 3C) and followed the kinetic of repopulation in the PB of the hosts exploiting the CD45.2 phenotype of the donor cells (Figure 3D). *Prep1^i/i^* cells generated less mature hematopoietic cells in PB compared to wt controls at all time points (Figure 3D). This agrees with the decrease of both mature myeloid and lymphoid cells in the PB of *Prep1^i/i^* recipients [24]. Twenty weeks after transplantation, we analyzed the repopulation efficiency of *Prep1^i/i^* v wt HSCs by counting the number of repopulating units (see Methods). This revealed a dramatic (86%) decrease in the ability of *Prep1^i/i^* HSCs to repopulate the entire hematopoietic system (Figure 3E). We also measured the ability of FL *Prep1^i/i^* HSCs to give rise to new stem cells, investigating the donor-derived HSCs compartment in the host. Again, the number of *Prep1^i/i^*-derived RUs dropped compared to wt controls (Figure 3F). The progenitor compartments were also negatively affected by the absence of Prep1 in repopulated mice (Table S1). The time of 20 weeks after transplantation, chosen to assess PB reconstitution, does not conflict with malignant transformation occurring in *Prep1^i/i^* mice at least 7–8 months after transplantation [21].

**Figure 4:**
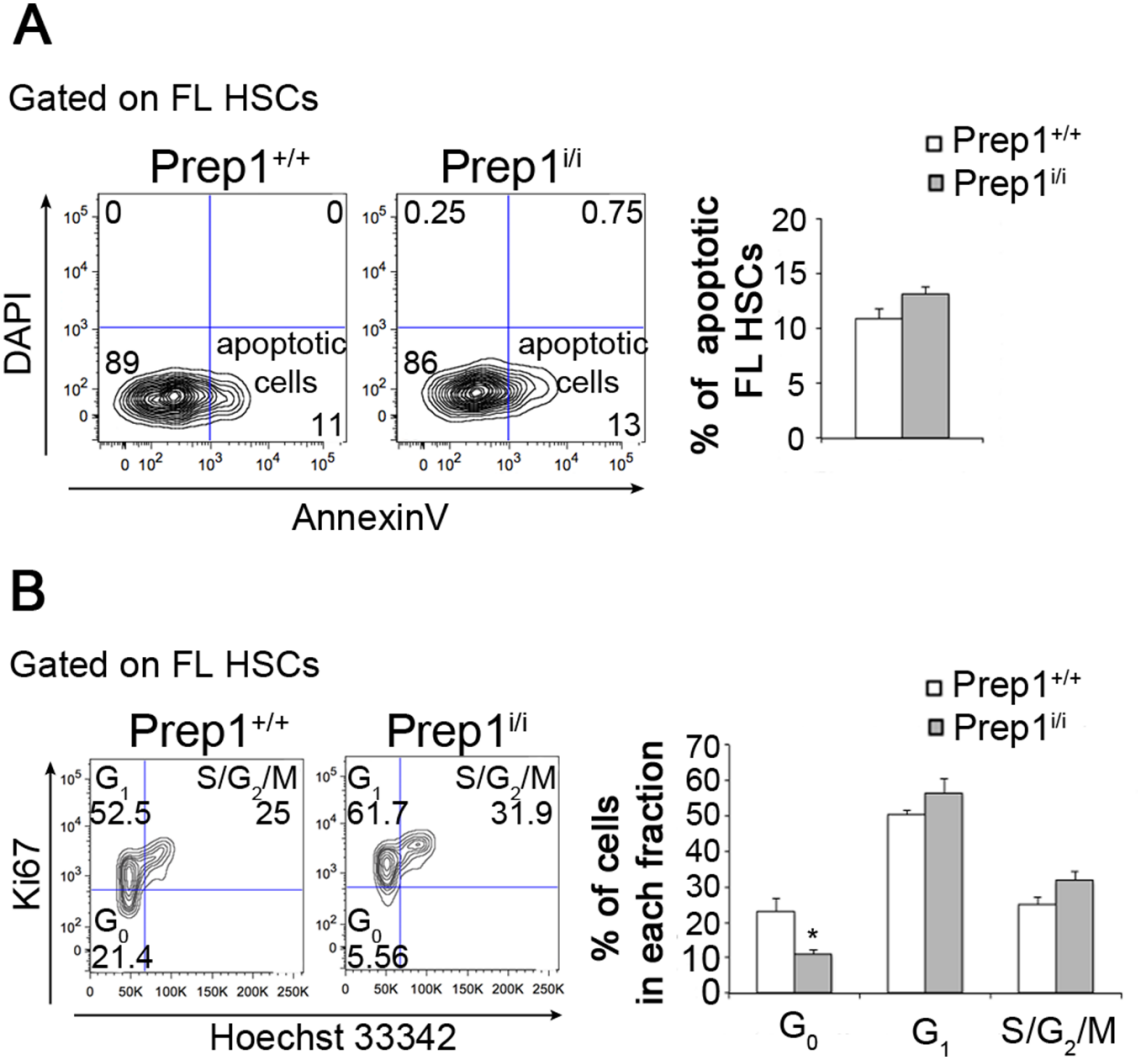
Prep1 influences HSC quiescent pool rather than HSCs apoptosis. (A) Representative FACS contour plots to identify apoptotic *Prep1^+/+^* and *Prep1^i/i^* HSCs are shown on the left. The represented plots refer to L^−^S^+^K^+^CD150^+^ gate and numbers in the FACS plots indicate the percentage of cells in parental gates. On the right, the graph represents the mean of apoptotic HSCs (Annexin^+^ DAPI^−^) (n = 3; p = not significant). (B) Representative FACS contour plots to identify the cell cycle distribution of *Prep1^+/+^* and *Prep1^i/i^* HSCs are shown on the left. The represented plots refer to L^−^S^+^K^+^CD150^+^ gate and numbers in the FACS plots indicate the percentage of cells in parental gates. On the right, the graph represents the mean of G0 (Ki67^−^Hoechst^l^°^w^), G1 (Ki67^+^Hoechst^l^°^w^) and S/G2/M (Ki67^+^Hoechst^hi^) HSCs (n = 3; p = not significant) (n = 3; G0 p = 0.02; G1 and S/G2/M p = not significant).

**Figure 5:**
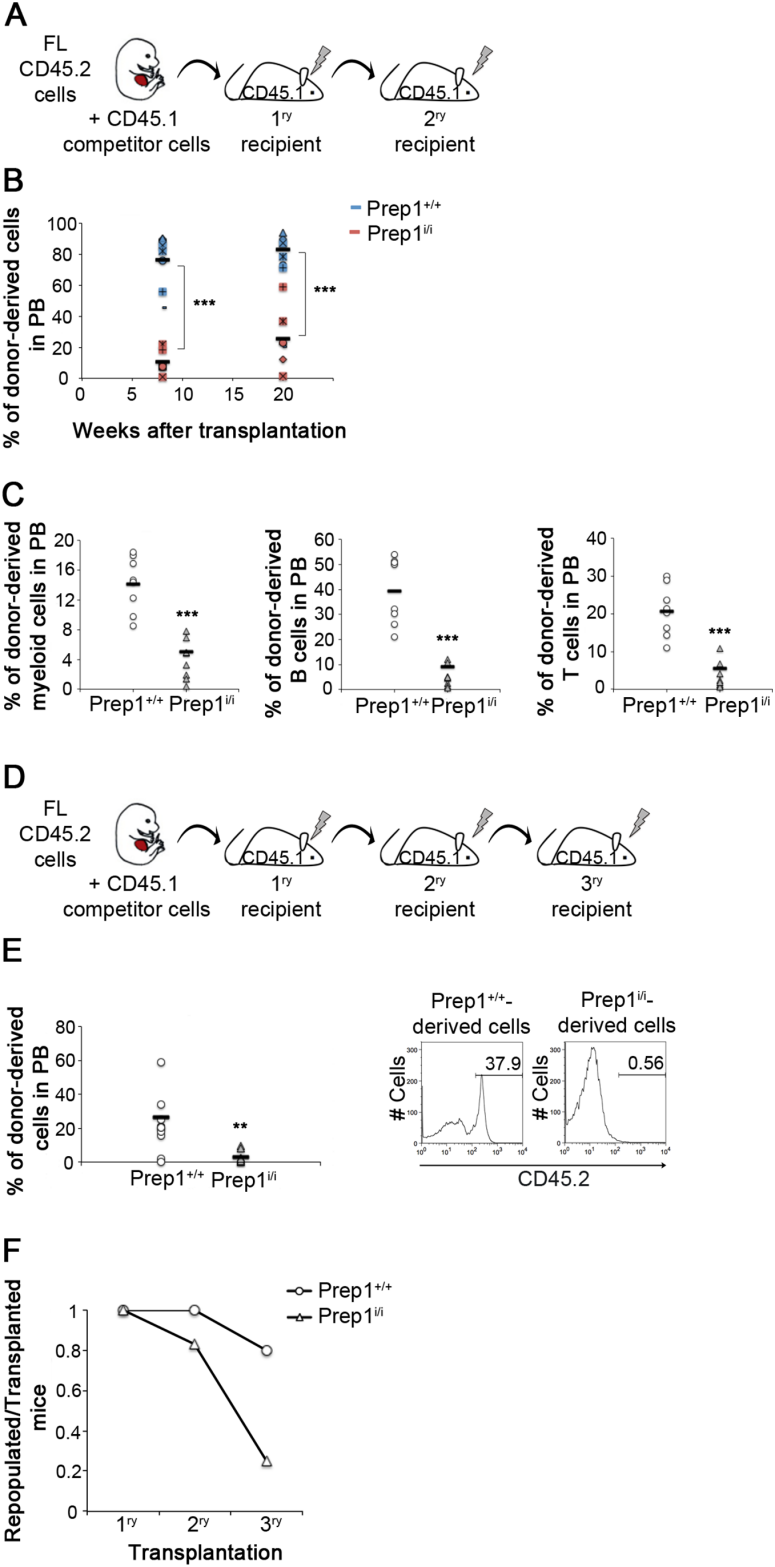
Prep1^i/i^ HSCs undergo exhaustion faster than wt in serial transplantation assay. (A) 2×10^6^ unfractionated BM cells from primary recipients were injected into secondary hosts. Primary recipients had received 1×10^6^ unfractionated CD45.2^+^ FL cells together with 1×10^6^ unfractionated CD45.1^+^ wt BM cells (chimerism shown in FigureS4). (B) PB analyses of secondary recipients were performed 8 and 20 weeks after transplantation to detect CD45.2^+^ cells. Each recipient is depicted with a separate symbol, blue and red colors indicating *Prep1^+/+^* and *Prep1^i/i^* reconstituted mice, respectively. Black bars represent the mean values in *Prep1^+/+^* or *Prep1^i/i^* reconstituted mice (n = 8 for each genotype at both time points; p-value<0.001 at both time points). (C) 20 weeks after transplantation, PB of secondary hosts was analysed for donor-derived myeloid cells (CD45.2^+^Gr1^+^Mac1^+^; p = 0.000017), B lymphoid cells (CD45.2^+^B220^+^; p = 0.000007) and T lymphoid cells (CD45.2^+^CD3^+^; p = 0.000017). Black bars represent mean values (n = 8 for each genotype). (D) Tertiary transplantations were performed injecting 2×10^6^ BM cells from secondary recipients into tertiary hosts. (E) PB of tertiary hosts was analyzed 20 weeks after transplantation to detect CD45.2^+^ cells. Black bars represent the mean values (n = 10 for *Prep1^+/+^* and n = 8 for *Prep1^i/i^*; p-value = 0.007). On the right, representative FACS histograms obtained for CD45.2^+^ cells of control and mutant tertiary recipients. (F) The ratio between the number of positively repopulated mice (CD45.2^+^ cells >2%) and transplanted mice is represented for primary, secondary and tertiary transplantations for both genotypes (primary transplantation n = 10 for each genotype; secondary transplantation n = 8 for each genotype; tertiary transplantation n = 10 for *Prep1^+/+^* and n = 8 for *Prep1^i/i^*).

Functional impairment or decreased HSCs number in Prep1-deficient FLs might be due to increased apoptosis. However, the experiment shown in figure 4A rules out this hypothesis since wt and *Prep1^i/i^* HSCs displayed comparable apoptosis level as measured by Annexin V binding. Nonetheless, when we moved to the analysis of the proliferative state of HSCs, we noticed a slight increase in the percentage of *Prep1^i/i^* HSCs in G1 and S-G2-M phases compared to wt and, importantly, a statistically significant 50% reduction in the Prep1 deficient G0 quiescent pool (Figure 4B). The depletion of the G0 pool in the stem cell compartment in the absence of Prep1 indicates that the functional defects observed in hypomorphic FLs might depend on a deregulated balance between quiescent and proliferative states which may lead to the exhaustion of the stem cell pool. To verify this hypothesis, we tested the self-renewal ability of fetal HSCs through serial transplantations into ablated hosts (Figure 5A–5D). BM cells from *Prep1^i/i^* transplanted mice were less efficient than wt in repopulating PB in secondary recipients (Figure 5B). 25% of *Prep1^i/i^* transplanted mice (2/8) showed no reconstitution at all after long-term repopulation. Eight weeks after secondary transplantation, reconstitution by donor cells was analyzed with respect to the myeloid, B-lymphoid and T-lymphoid lineages (Figure 5C). *Prep1^i/i^* donor cells were much less efficient than wt littermates in generating all the lineages, with a 30% decrease of myeloid CD45.2^+^Gr1^+^Mac1^+^ and 75% reduction of both CD45.2^+^B220^+^ and CD45.2^+^CD3^+^ lymphoid cells (Figure 5C).

When we transferred BM cells from secondary to tertiary recipients (Figure 5D), *Prep1^i/i^* derived cells lost their repopulation capacity as only a minor fraction of the tertiary transplanted hosts (2/8) showed a low repopulation activity (Figure 5E–F). In summary, *Prep1^i/i^* HSCs initially repopulate primary recipients as wt controls. However, in secondary and tertiary recipients the *Prep1^i/i^* stem cell pool decreases tending to premature exhaustion (Figure 5F).

To directly support the above conclusion, we re-transplanted primary recipient’s BM cells after readjusting the CD45.2/CD45.1 ratio in donor cells by FACS sorting (six mice for wt and 2 for hypomorphic). The result was unequivocal with a mean 42 v 5% donor-derived cells in wt and *Prep1^i/i^* secondarily transplanted mice (Figure S2A). Similarly, in PB donor-derived myeloid, B-lymphoid and T lymphoid cells were dramatically reduced in *Prep1^i/i^* compared to wt reconstituted mice (Figure S2B–D).

### Prep1 modulates the interferon signaling pathway in FL HSCs

**Figure 6:**
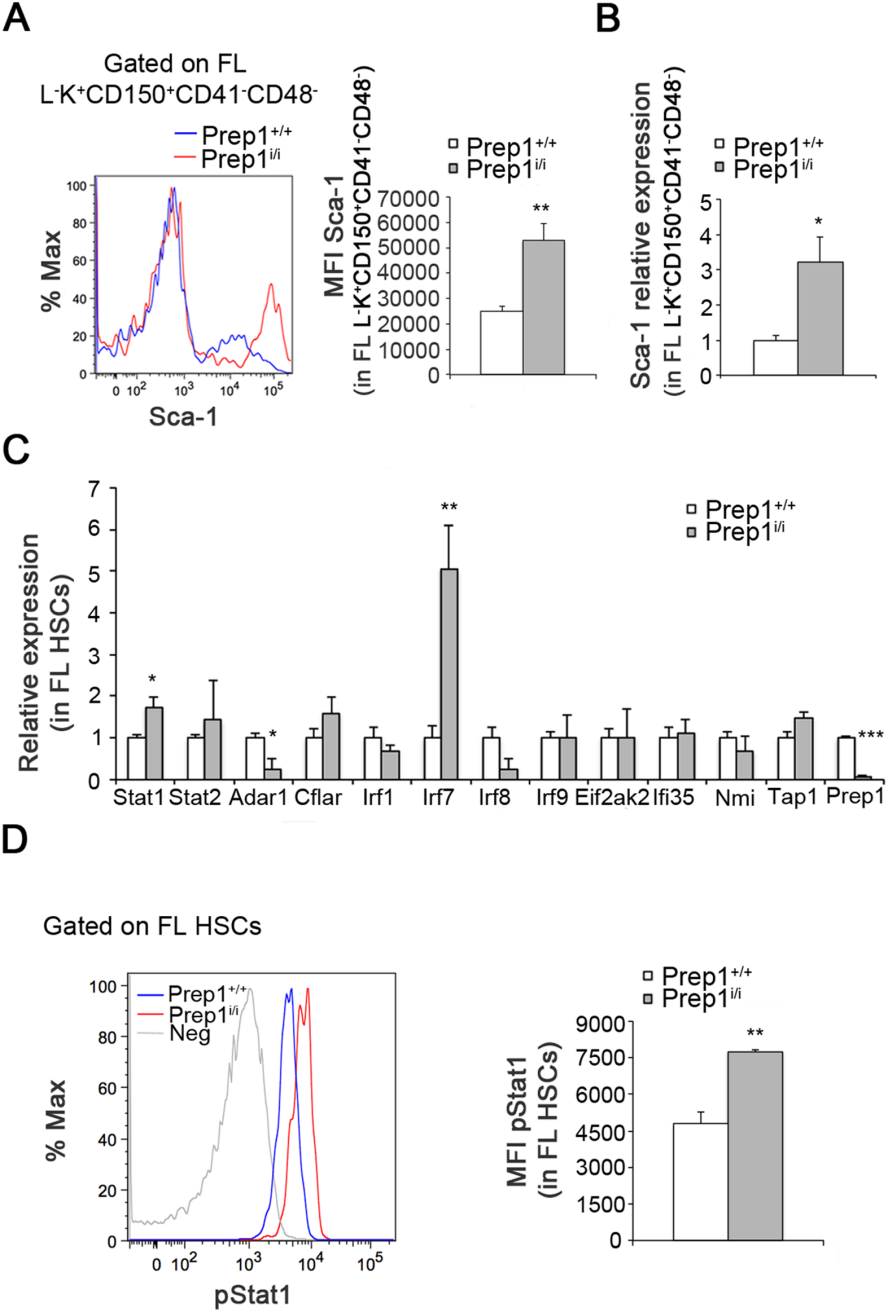
Prep1 modulates the IFN signaling pathway of FL HSCs. (A) The expression of Sca-1 on the cell surface of *Prep1^+/+^* and *Prep1^i/i^* L^−^K^+^CD150^+^CD48^−^CD41^−^ is quantified by mean fluorescent intensity (MFI). The representative FACS histogram on the left refers to L^−^K^+^CD150^+^CD48^−^CD41^−^ gate (n = 8 for each genotype; p = 0.003). (B) qRT-PCR was performed on sorted *Prep1^+/+^* and *Prep1^i/i^* L^−^S^+^K^+^CD150^+^CD48^−^CD41^−^ cells to detect Sca1 transcripts. Values are normalized on GAPDH expression (n = 4 for each genotypes; p = 0.02). (C) The histograms show the expression of the indicated genes in sorted *Prep1^+/+^* and *Prep1^i/i^* L^−^S^+^K^+^CD150^+^CD48^−^CD41^−^ cells as detected by qRT-PCR. (n = 4 for each genotypes; Stat1 p = 0.02; Adar1 p = 0.02; Irf7 p = 0.003; Prep1 p = 0.00002). (D) The representative FACS histogram on the left refers to the L^−^S^+^K^+^CD150^+^ gate. The negative control (grey histogram) tracing represents a sample stained for all the surface markers plus the fluorochrome-conjugated secondary antibody used to detect pStat1, but without the pStat1 antibody. (n = 3 for each genotype; p = 0.01). The phosphorylation of Stat1 (pStat1) was analyzed by MFI and quantitated on the right.

The reduced quiescent stem cell pool and the premature exhaustion of HSCs in the *Prep1^i/i^* FL suggest that Prep1 regulates self-renewal by controlling the quiescence/proliferation balance. To gain insight into the signaling cascades and the molecular mechanisms regulated by Prep1 in FL HSCs, we investigated the increased expression of the surface marker Sca-1 in L^−^S^+^K^+^ cells (Figure 1C). Sca-1 is known to regulate the repopulation activity and self-renewal of HSCs in both FL and BM [33], [34]. We measured in *Prep1^i/i^* FL HSCs a 2-fold increase in Sca-1 antigen compared to wt control littermates (Figure 6A) and a comparable increase in its transcript level (Figure 6B). Since Sca-1 is an IFN-inducible gene [35] and is upregulated by IFNs in FL HSCs [33], we investigated whether other components of this signaling pathway are perturbed in *Prep1^i/i^* HSCs measuring the mRNA level of a panel of genes implicated in the IFN response (Figure 6C). We observed an increase in the expression of Stat1, Stat2 and Irf7 in *Prep1^i/i^* HSCs compared to wt littermates. Moreover we also noticed a statistically significant reduction in the expression of Adar1 in *Prep1^i/i^* cells.

**Figure 7:**
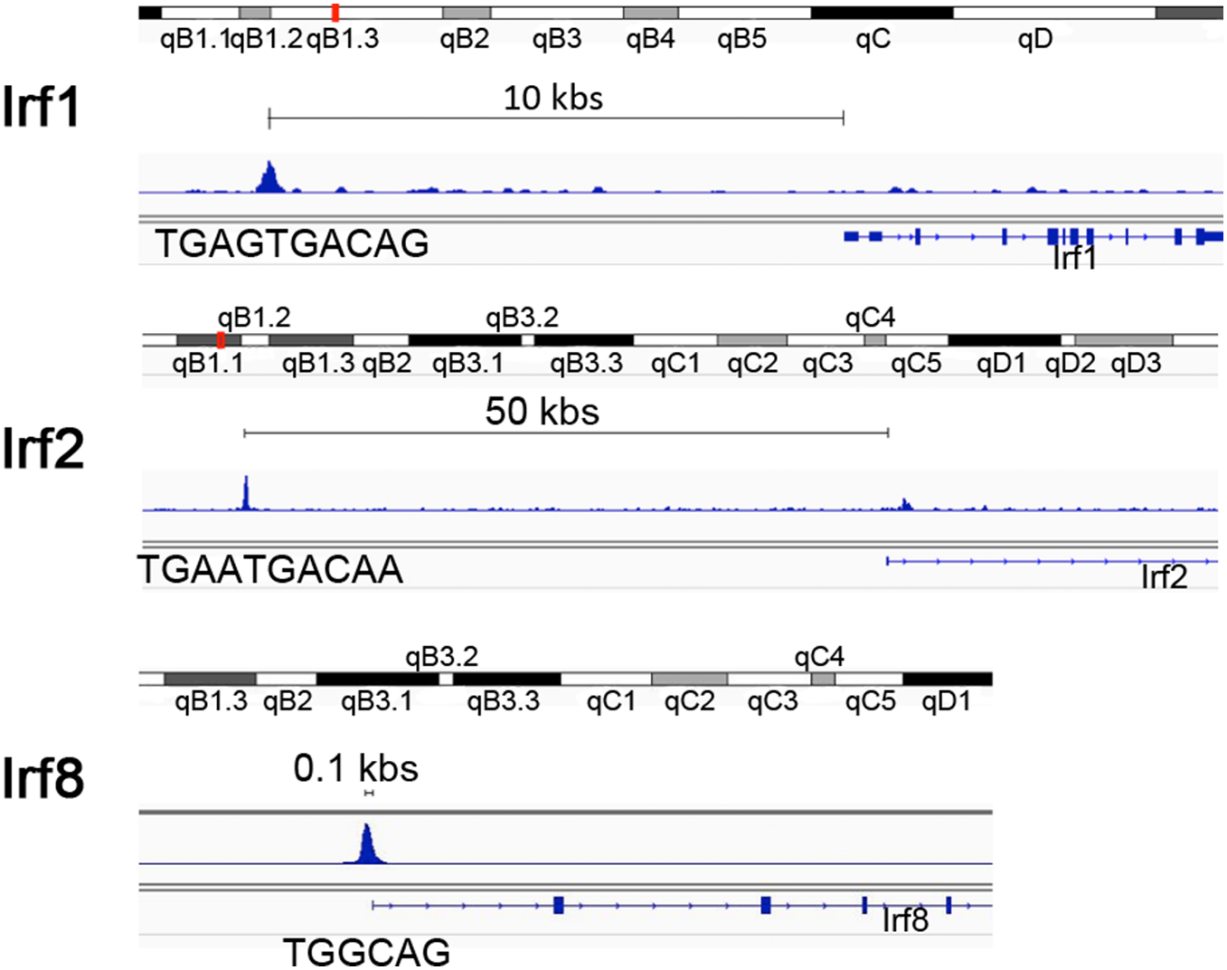
Prep1 ChIP-seq tracing of the mouse embryo Irf1, Irf2 and Irf8 genes. ChIPseq tracing from the m9 mouse genome. In the *Irf1* gene a Prep1 peak is present about 10 Kbp upstream of the promoter: the peak is centered around the TGAGTGACAG Prep1 consensus [36]. In the *Irf2* gene, the peak is present 50 Kbp upstream of the promoter and is centered around a similar TGAATGACAA Prep1 consensus [36]. In the *Irf8* gene, the peak is right at the promoter, 100 bp distance, centered around a less frequent hexameric consensus, TGGCAG [36]. Chromatin immunoprecipitation was performed on E11.5 mouse embryos [36] and was confirmed in ES cells.

Furthermore a ChIP sequencing analysis performed on mouse embryos [36] (and embryonic stem cells) revealed that Irf1, Irf2 and Irf8 are direct Prep1 targets (Figure 7). The peaks, having a highly significant intensity, are located about from Kb to few bp (*Irf8*) before the transcription start site of the genes. These data together with the increased expression of signaling mediators like Stat1, Stat2 and of *Irf7* and the decrease of the suppressor of the IFN response (Adar1) indicate that Prep1 regulates the IFN pathway. To corroborate the involvement of STATs, we investigated the activation of Stat1 comparing its phosphorylation state in wt and *Prep1^i/i^* HSCs by flow cytometry and observed a statistically significant 1.6 fold-increase in pStat1 in *Prep1^i/i^* HSCs (Figure 6D).

**Figure 8:**
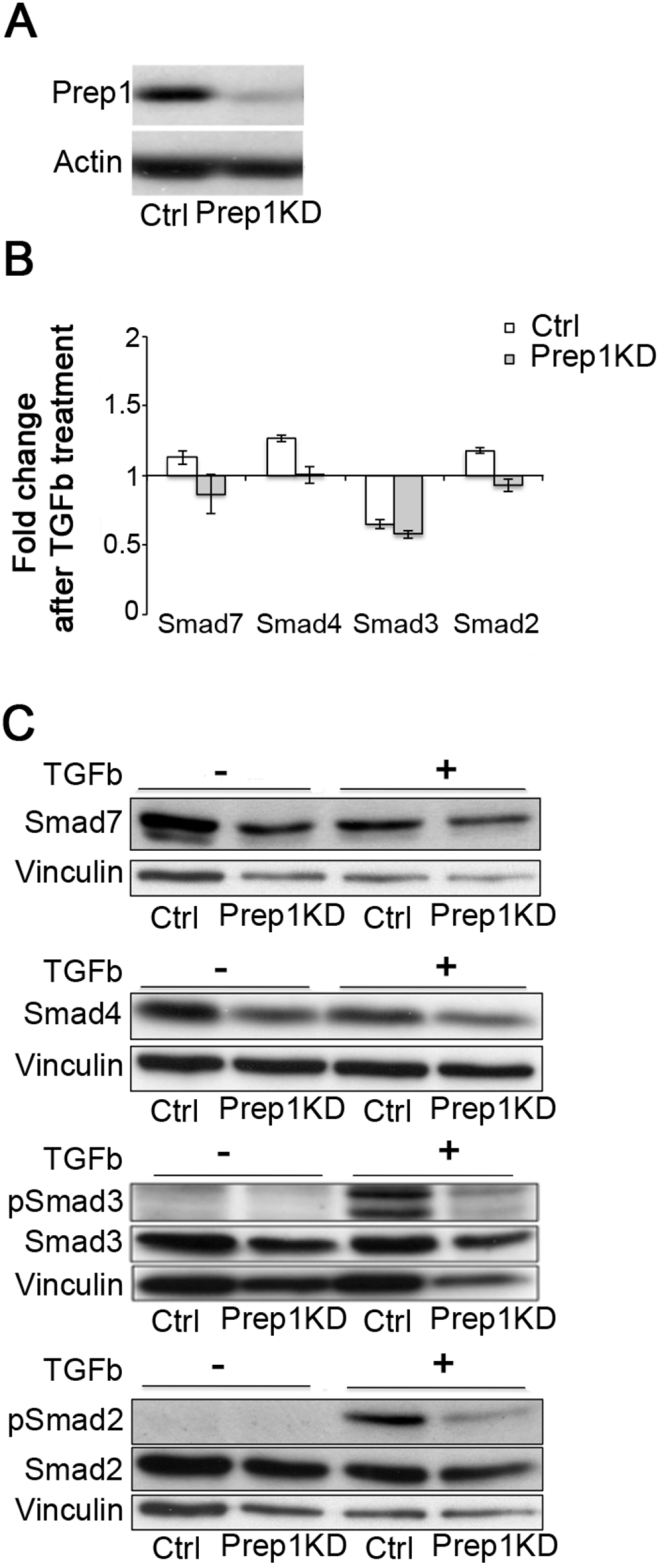
Prep1 does not affect Smads expression but modulates their phosphorylation in the EML1 progenitor cell line. (A) Prep1 knock-down (KD) was assessed by Immuno-blotting analysis. Actin was used as loading control. (B–C) Prep1 KD or control EML1 cells were incubated 4 h with (+) or without (−) TGFβ (10 ng/ml). (B) Histograms show fold induction of the indicated transcripts in Prep1 KD or control EML1 cells as measured by qRT-PCR. The data represent 2 independent experiments. (C) Smads and their phosphorylated forms (as indicated) were detected by Western blot analyses. Vinculin was used as loading control. The data were reproduced in 2 independent experiments.

Moreover, since the ablation of Pbx1 in adult HSCs influences the TGFbeta pathway [37], we investigated whether Prep1 acts in a similar way in FLs. To this aim, we knocked down (KD) Prep1 by shRNA in the EML1 progenitors cell line and assessed their responsiveness to TGFbeta. We found no significant alteration in SMAD levels at both RNA and protein level (Figure 8B–C). However, the phosphorylation of Smad2 and Smad3 was reduced in Prep1^KD^ (Figure 8C). This suggests that Prep1 may partly act modulating TGFbeta.

We next investigated the status of the IFN pathway in *Prep1^i/i^* HSCs in the adult wt environment after transplantation. Neither the activation of Stat1 (pStat1) (Figure S3A) nor the overexpression of Sca-1 was reproduced in the BM niche of transplanted mice (Figure S3B). Moreover, since Sca-1 overexpression is a distinguishing marker of proliferative v. quiescent HSCs [34], we investigated the cell cycle status of these cells. In the adult environment, *Prep1^i/i^* and wt HSCs did not show any statistically significant difference in the quiescent G0 stem cell pool (Figure S3C). This indicates that the effect of Prep1 on the Stat1/Sca1 proliferative pathway is specific to fetal cells and may be dispensable during adult hematopoiesis.

## Discussion

We have shown that Prep1 controls the number and function of HSC during mouse embryo development. Prep1 regulates HSC self-renewal since *Prep1^i/i^* cells are deficient in generating the stem cell compartment upon transplantation into ablated hosts (Figure 3) and undergo faster exhaustion upon serial transplantation (Figure 5).

These results are coherent with the role in hematopoiesis of other TALE family members: Pbx1 and Meis1. Pbx1 regulates transcription mostly as a dimer with Prep1 or Meis1. In a mixed cells population Meis1-Pbx1 complex mainly associated with intragenic regions of developmental genes, whereas Prep1-Pbx1 bound promoters of genes involved in basic cell functions [36]. Pbx1 and Meis1 play an important role in the hematopoietic compartment as both FL *Pbx1^−/−^* [38] and *Meis1^−/−^* HSCs [39], [40] are inefficient in establishing multi-lineage hematopoiesis when tested in transplantation experiments. Moreover, Pbx1 regulates BM HSC self-renewal by maintaining the quiescent stem cell pool [37]. Adult *Pbx1^−/−^* HSCs show a reduced G0 fraction and undergo exhaustion faster than wt. Pbx1 also has a lineage-specific role preventing myeloid differentiation and maintaining lymphoid potential [41]. As Prep1 is the most common partner of Pbx1 [36], the phenotype of *Prep1^i/i^* HSC may be similar to *Pbx1^−/−^*. However, CMPs and CLPs frequency in the *Pbx1^−/−^* background is different from *Prep1^i/i^*, where the frequency of lymphoid progenitors is drastically increased. Moreover, whereas Meis1 and Pbx1 act in concert to transcriptionally modulate TGFbeta response [37], [42], Prep1 is mainly implicated in the regulation of the IFN-response pathway and may affect the TGFbeta pathway in the adult. The phenotypes may also depend on additional non-transcriptional effects of Prep1 as both Meis1 and Pbx1 are decreased in P*rep1^i/i^* FL [20].

In this paper we also show that Prep1 regulates the cell cycle state of the FL HSCs, maintaining cells in the G0 phase (Figure 4). Embryonic FL HSCs are mainly proliferating cells in comparison with the quiescent adult BM HSCs. At E14.5, FL HSCs cycle every 48 hours [43], while adult HSCs divide every 7 weeks [44], [45]. As a consequence, G0 phase FL HSCs cannot aptly be considered the fetal counterpart of the adult HSCs which represent a “dormant” pool. Rather, the prolonged permanence in G0 is a requirement to assure that the modality of cell division corresponds to biological necessities. Furthermore, in terms of cell cycle the transit through G0 and the prolonged cell cycle are peculiarities of HSCs versus progenitors cells [43]. In this view, Prep1 might act as an intrinsic factor necessary at mid-gestation to regulate the cell cycle of HSCs and to preserve their self-renewal ability.

In homeostatic conditions quiescent BM HSCs enter the cell cycle dividing preferentially by asymmetric cell division with the aim to produce at the same time mature cells and to maintain a stem cell pool [46]–[48], FL HSCs mainly divide by symmetric self-renewing divisions in order to expand the HSC pool [49], [50]. We show that, in E14.5 FLs, Prep1 deficiency causes a reduction in the number of HSCs and an increase in multipotent progenitors identified as L^−^S^+^K^+^ cells. The inverse correlation between stem and progenitor cells *Prep1^i/i^* embryos might be caused by a change in the balance between symmetric and asymmetric cell divisions skewed towards the asymmetric mode, leading to a reduced stem cell pool and the increased progenitor compartment.

In addition, the increased cycling shown by *Prep1^i/i^* fetal HSCs positively correlates with the role of Prep1 as tumor suppressor. Indeed *Prep1^i/i^* FL cells cause lymphomas when transplanted in hosts after a long latency [21] and the deregulated cell cycle might contribute to make these cells prone to malignant transformation.

Furthermore, *Prep1^i/i^* FL HSCs display an activated IFN-induced response as demonstrated by the activation of STAT1, increased IFN-induced transcripts and decreased Adar1 (Figure 6). This feature positively correlates with the reduced number of HSCs in *Prep1^i/i^* FLs. IFN signaling has been shown to regulate HSCs properties in both adult and fetal tissues. Indeed, treatment of adult HSCs with IFNα induces their proliferation with the concomitant decrease of the G0 pool causing HSCs exhaustion, an effect mediated by activation of STAT1 and upregulation of Sca-1 [34]. In addition, Adar1 deaminase suppresses IFN signaling and is strongly down-regulated in FL HSCs [33]. Similarly to *Prep1^i/i^* HSCs, *Adar1^−/−^* FL HSCs upregulate IFN inducible genes (Sca-1, Stats and Irfs) and show a diminished number of HSCs [33].

Although not yet directly tested, it is tempting to speculatre that the Prep1-absence-dependent activation of IFN response is connected to DNA damage accumulation. The induction of DNA damage in human and mouse cells triggers Atm-mediated DNA damage response that, in turn, through the activation of NFKB leads to the expression of IFN and IFN-induced genes such as *Irf1* and *Irf7* [51]. Interestingly, Prep1 acts as a tumor suppressor in maintaining genome stability in cells [22]. FL HSCs are exposed to proliferative stress with accumulation of mutations and genome instability *per se,* and *Prep1^i/i^* FL cells show DNA damage accumulation [22]. We speculate that the activation of the IFN response in *Prep1^i/i^* HSCs could possibly be induced by intrinsic DNA damage due to Prep1 deficiency. The direct binding of Prep1 to three interferon-response genes (*Irf1*, *Irf2*, *Irf8*), the increased expression of Irf7 and repression of Irf8 support the idea that Prep1 regulates the IFN pathway. These factors orchestrate the pathway and affect stem and progenitors cell proliferation and differentiation [52].

The present work also shows that the Stats/Sca1 proliferative axis is is no longer activated in *Prep1^i/i^* HSCs isolated from adult transplanted BM niche (Figure S3). This is not a unique case in hematopoietic development as *Mll^−/−^* mice lose quiescent G0 HSCs and show repopulation defects. Importantly, the Mll-dependent self-renewal defect is much stronger in the FL than in the adult BM niche [53]. The dual behavior of hypomorphic cells suggests that an embryonic niche-specific signal is required for the *Prep1^i/i^* fetal phenotype and possibly suggests different proliferative cues in FLs and adult quiescent BM niche. An additional regulator of HSC activity missing in *Prep1^i/i^* cells might be the TGFbeta-dependent activation of Smad2/Smad3, in agreement with the *Pbx1^−/−^* phenotype [37].

## Conclusions

In this work, we demonstrate that the homeodomain transcription factor Prep1 controls the number and the biological activities of fetal HSCs. In particular, we show that Prep1 regulates the self-renewal ability and the cell cycle distribution of HSCs during fetal hematopoiesis by regulating the activation of Stat1, Sca-1 via the IFN-dependent proliferative pathway.

## Supporting Information

Figure S1 **The HSC-enriched population L^−^S^+^K^+^AA4.1^+^ is affected by the absence of Prep1.** (A) Representative FACS dot plots to identify L^−^S^+^K^+^AA4.1^+^ cells in *Prep1^+/+^* and *Prep1^i/i^* FLs. The plots show AA4.1^+^ cells in the L^−^S^+^K^+^ population and the numbers indicate their percentage in the parental gate. (B) Graphs describe the percentage (left) and absolute numbers (left) of the L^−^S^+^K^+^AA4.1^+^ population (n = 8 for *Prep1^+/+^* and n = 12 for *Prep1^i/i^* FLs in both graphs; % L^−^S^+^K^+^AA4.1^+^ p = not significant; # L^−^S^+^K^+^AA4.1^+^ p = 0.02).
(TIF)

Figure S2 ***Prep1^i/i^*** **cells in a readjusted ratio with competitors show defective repopulation upon secondary transplantation.** 1×10^6^ *Prep1^+/+^* or *Prep1^i/i^* CD45.2^+^ cells sorted from primary recipients were mixed with 3×10^5^ sorted CD45.1^+^ competitor cells and transplanted into lethally irradiated secondary recipients. (A) To detect donor-derived (CD45.2^+^) cells, PB analyses of secondary recipients were performed 16 weeks after transplantation. White diamonds and grey triangles indicate *Prep1^+/+^* and *Prep1^i/i^* reconstituted mice, respectively. Black bars represent the mean of CD45.2^+^ cells in *Prep1^+/+^* or *Prep1^i/i^* reconstituted mice (n = 6 for *Prep1^+/+^* and n = 2 for *Prep1^i/i^*. p = 0.02). (B-C-D) 16 weeks after transplantation, PB of secondary hosts was analysed (B) for the presence of donor-derived myeloid cells (CD45.2^+^Gr1^+^Mac1^+^; p-value = 0.008), (C) donor-derived B lymphoid cells (CD45.2^+^B220^+^; p-value = 0.04) and (D) donor-derived T lymphoid cells (CD45.2^+^CD3^+^; p-value = 0.02). Black bars represent mean values (n = 6 for *Prep1^+/+^* and n = 2 for *Prep1^i/i^*).
(TIF)

Figure S3 **The IFN-induced signaling pathway is not induced in the absence of Prep1 in the adult BM niche.** (A–C) pStat1, Sca1 and cell cycle distribution were analyzed in *Prep1^+/+^* and *Prep1^i/i^*-derived HSCs in the BM of primary transplanted mice that received 1×10^6^ unfractionated FL cells together with 1×10^6^ unfractionated wt BM cells. Representative FACS plots are reported on the left and their quantifications on the right. (A) pStat1 intensity was evaluated by MFI. FACS plots are referred to CD45.2^+^L^−^S^+^K^+^CD150^+^ gate (n = 3 for each genotypes; p = not significant; representative of 3 independent experiments). (B) Sca-1 intensity was evaluated by MFI. FACS plots are referred to CD45.2^+^L^−^S^+^K^+^CD150^+^CD48^−^CD41^−^ gate (n = 4 for each genotypes; p = not significant; representative of 3 independent experiments). (C) Cell cycle distribution in G0, G1 and S/G2/M phases was evaluated for donor-derived HSCs by Ki67/Hoechst 33342 staining. FACS plots are referred to CD45.2^+^L^−^S^+^K^+^CD150^+^ gate (n = 5 for each genotypes; G0, G1 and S/G2/M p = not significant).
(TIF)

Figure S4 **Chimerism in primary recipients of the serial transplantation experiment.** Primary recipients received 1*10^6^ unfractionated CD45.2 FL cells from *Prep1^+/+^* or *Prep1^i/i^* embryos together with 1*10^6^ unfractionated CD45.1 BM cells. Percentages of donor-derived cells (on the left) were calculated on FACS data (on the right) obtained from BMs of transplanted mice 20 weeks after transplantation.
(TIF)

Table S1 **Progenitor compartments are affected in transplanted hosts in the absence of Prep1.** 2000 LSK cells purified form *Prep1^+/+^* or *Prep1^i/i^* FLs were transplanted in competition with 1×10^6^ BM cells into lethally irradiated CD45.1 recipients. LSK, CLP and CMP RUs (±SEM) are calculated in the BM of transplanted primary recipients (see Methods). (RU = repopulating units; LSK = Lin^−^Sca-1^+^cKit^+^ cells; CLP = common lymphoid progenitors; CMP = common myeloid progenitors).
(DOCX)
