# Supplementary figures and images for: Prep1 (pKnox1) Regulates Mouse Embryonic HSC Cycling and Self-Renewal Affecting the Stat1-Sca1 IFN-Dependent Pathway
Source: PLoS One. 2014 Sep 18;9(9):e107916. doi: 10.1371/journal.pone.0107916 (PMC4169458; doi:10.1371/journal.pone.0107916)

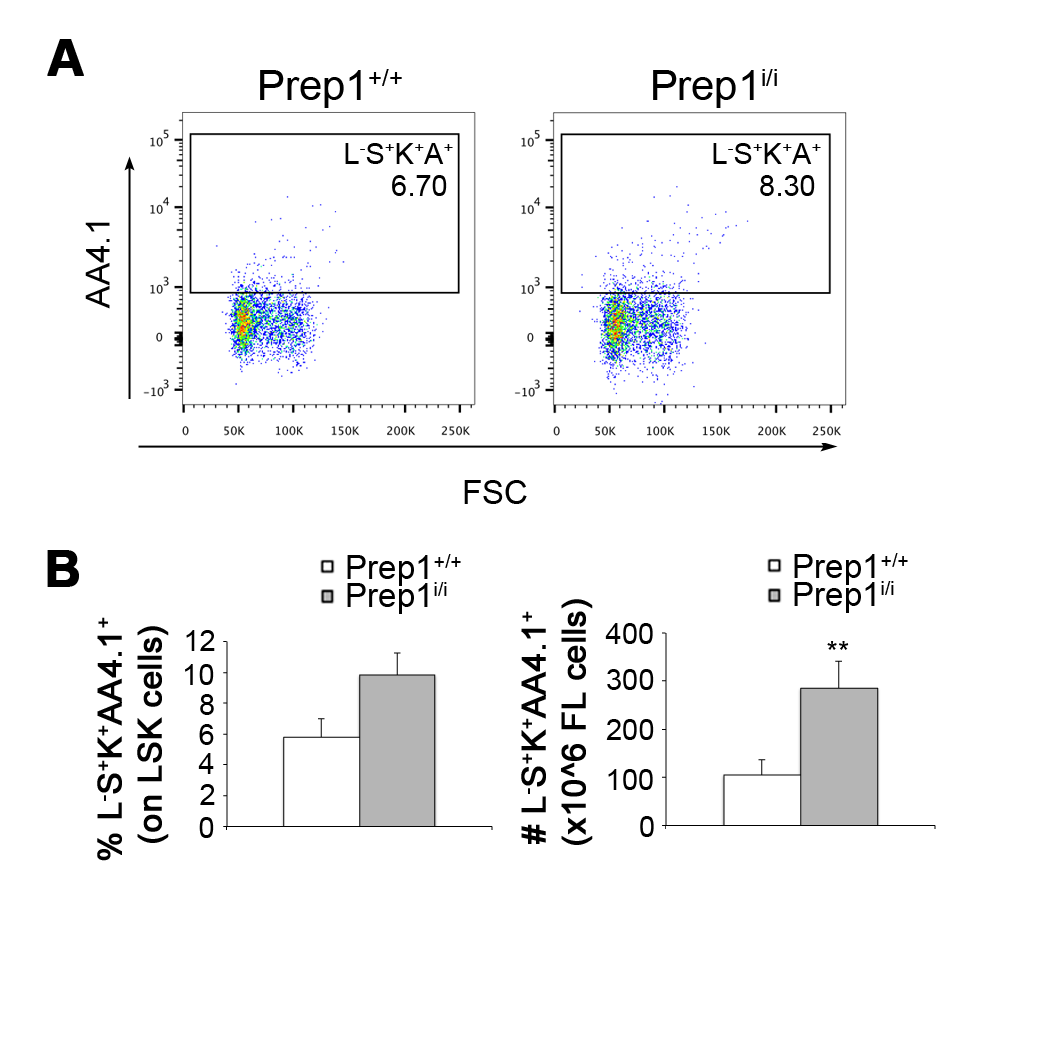

Supplement: Figure S1 — The HSC-enriched population L−S+K+AA4.1+ is affected by the absence of Prep1. (A) Representative FACS dot plots to identify L−S+K+AA4.1+ cells in Prep1+/+ and Prep1i/i FLs. The plots show AA4.1+ cells in the L−S+K+ population and the numbers indicate their percentage in the parental gate. (B) Graphs describe the percentage (left) and absolute numbers (left) of the L−S+K+AA4.1+ population (n = 8 for Prep1+/+ and n = 12 for Prep1i/i FLs in both graphs; % L−S+K+AA4.1+ p = not significant; # L−S+K+AA4.1+ p = 0.02). (TIF) [file pone.0107916.s001.tif]

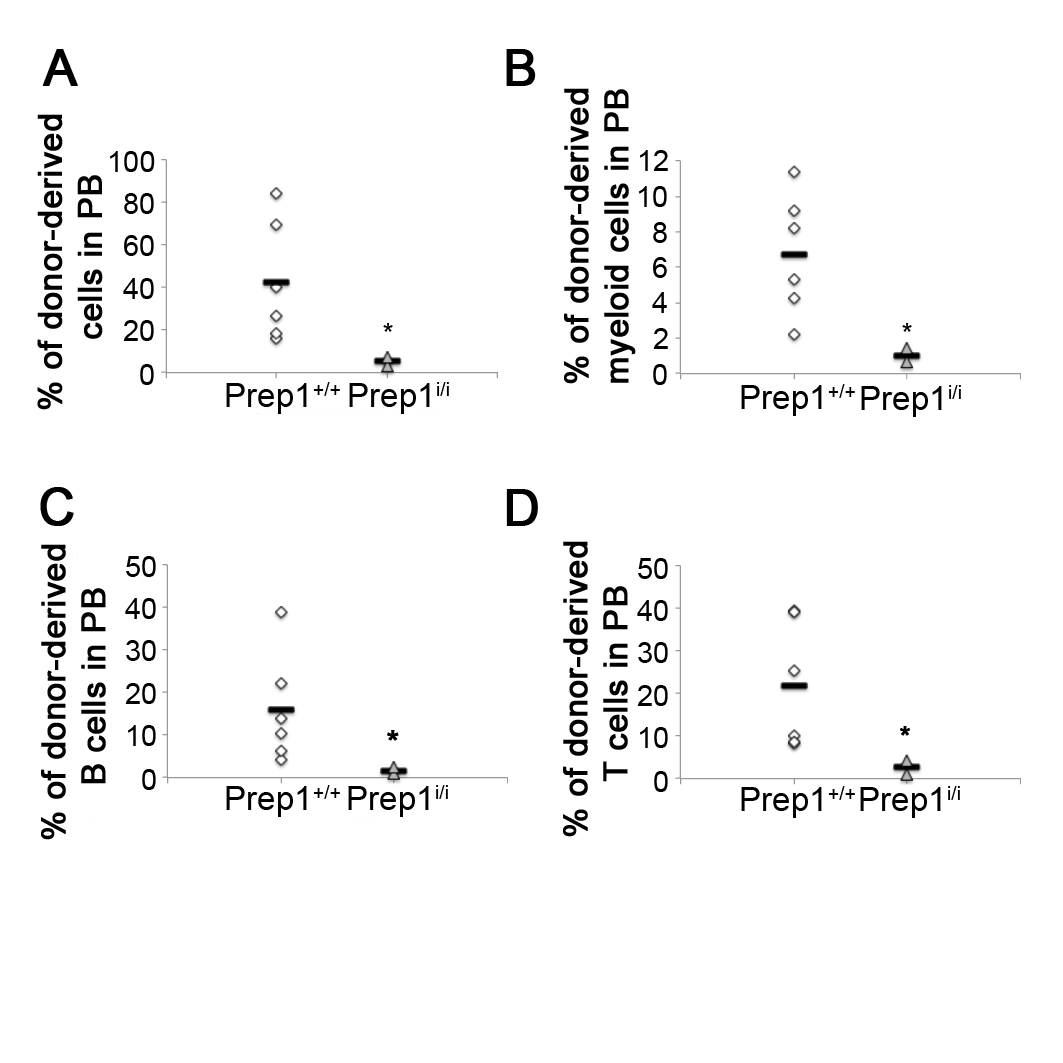

Supplement: Figure S2 — Prep1i/i cells in a readjusted ratio with competitors show defective repopulation upon secondary transplantation. 1×10^6^ Prep1+/+ or Prep1i/i CD45.2+ cells sorted from primary recipients were mixed with 3×10^5^ sorted CD45.1+ competitor cells and transplanted into lethally irradiated secondary recipients. (A) To detect donor-derived (CD45.2+) cells, PB analyses of secondary recipients were performed 16 weeks after transplantation. White diamonds and grey triangles indicate Prep1+/+ and Prep1i/i reconstituted mice, respectively. Black bars represent the mean of CD45.2+ cells in Prep1+/+ or Prep1i/i reconstituted mice (n = 6 for Prep1+/+ and n = 2 for Prep1i/i. p = 0.02). (B-C-D) 16 weeks after transplantation, PB of secondary hosts was analysed (B) for the presence of donor-derived myeloid cells (CD45.2+Gr1+Mac1+; p-value = 0.008), (C) donor-derived B lymphoid cells (CD45.2+B220+; p-value = 0.04) and (D) donor-derived T lymphoid cells (CD45.2+CD3+; p-value = 0.02). Black bars represent mean values (n = 6 for Prep1+/+ and n = 2 for Prep1i/i). (TIF) [file pone.0107916.s002.tif]

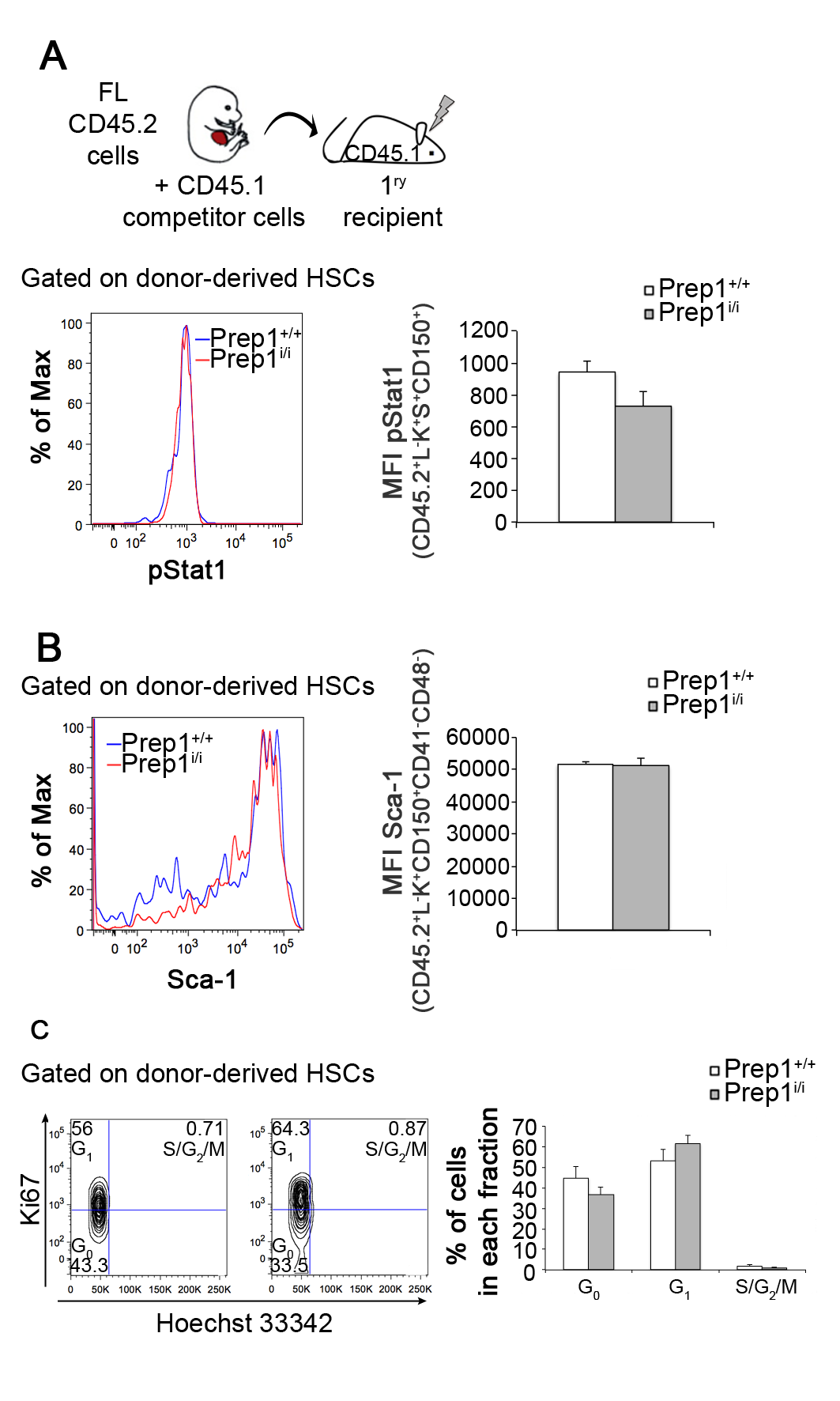

Supplement: Figure S3 — The IFN-induced signaling pathway is not induced in the absence of Prep1 in the adult BM niche. (A–C) pStat1, Sca1 and cell cycle distribution were analyzed in Prep1+/+ and Prep1i/i-derived HSCs in the BM of primary transplanted mice that received 1×10^6^ unfractionated FL cells together with 1×10^6^ unfractionated wt BM cells. Representative FACS plots are reported on the left and their quantifications on the right. (A) pStat1 intensity was evaluated by MFI. FACS plots are referred to CD45.2+L−S+K+CD150+ gate (n = 3 for each genotypes; p = not significant; representative of 3 independent experiments). (B) Sca-1 intensity was evaluated by MFI. FACS plots are referred to CD45.2+L−S+K+CD150+CD48−CD41− gate (n = 4 for each genotypes; p = not significant; representative of 3 independent experiments). (C) Cell cycle distribution in G0, G1 and S/G2/M phases was evaluated for donor-derived HSCs by Ki67/Hoechst 33342 staining. FACS plots are referred to CD45.2+L−S+K+CD150+ gate (n = 5 for each genotypes; G0, G1 and S/G2/M p = not significant). (TIF) [file pone.0107916.s003.tif]

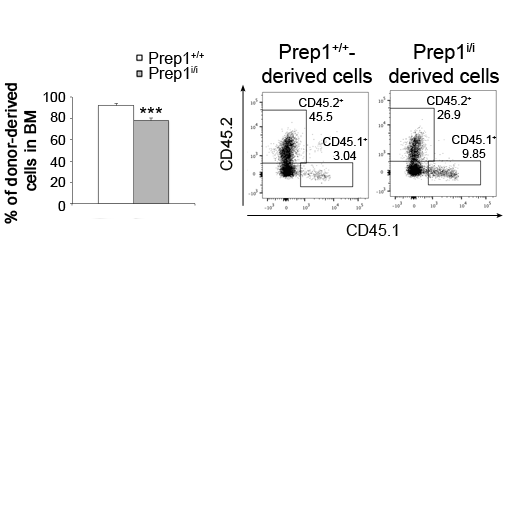

Supplement: Figure S4 — Chimerism in primary recipients of the serial transplantation experiment. Primary recipients received 1*106 unfractionated CD45.2 FL cells from Prep1+/+ or Prep1i/i embryos together with 1*106 unfractionated CD45.1 BM cells. Percentages of donor-derived cells (on the left) were calculated on FACS data (on the right) obtained from BMs of transplanted mice 20 weeks after transplantation. (TIF) [file pone.0107916.s004.tif]
