# Supplementary material for: Prep1 (pKnox1) Regulates Mouse Embryonic HSC Cycling and Self-Renewal Affecting the Stat1-Sca1 IFN-Dependent Pathway
Source: PLoS One. 2014 Sep 18;9(9):e107916. doi: 10.1371/journal.pone.0107916 (PMC4169458; doi:10.1371/journal.pone.0107916)
Supplement: Table S1 — Progenitor compartments are affected in transplanted hosts in the absence of Prep1. 2000 LSK cells purified form Prep1+/+ or Prep1i/i FLs were transplanted in competition with 1×10^6^ BM cells into lethally irradiated CD45.1 recipients. LSK, CLP and CMP RUs (±SEM) are calculated in the BM of transplanted primary recipients (see Methods). (RU = repopulating units; LSK = Lin−Sca-1+cKit+ cells; CLP = common lymphoid progenitors; CMP = common myeloid progenitors). (DOCX) [file pone.0107916.s005.docx]

|  | RU Prep1^+/+^ | RU Prep1^i/i^ | p-value |
| --- | --- | --- | --- |
| LSK | 0.093±0.028 | 0.015±0.0046 | 0.4 |
| CLP | 0.0018±0.0012 | 0.00012±0.000035 | 0.2 |
| CMP | 0.98±0.04 | 0.0037±0.0012 | 0.03 |
